# Epidemiology of ossification of the spinal ligaments and associated factors in the Chinese population: a cross-sectional study of 2000 consecutive individuals

**DOI:** 10.1186/s12891-019-2569-1

**Published:** 2019-05-25

**Authors:** Haifeng Liang, Guobing Liu, Shunyi Lu, Shuguang Chen, Dongjie Jiang, Hongcheng Shi, Qinming Fei

**Affiliations:** 1 Department of Orthopedic Surgery, Zhongshan Hospital, Fudan University, Building 1, 180 Fenglin Road, Shanghai, 200032 People’s Republic of China; 2 Department of Nuclear Medicine, Zhongshan Hospital, Fudan University, F B1, Building 16, 180 Fenglin Road, Shanghai, 200032 People’s Republic of China

**Keywords:** Whole spine, Computed tomography, Ossification, Spinal ligament, Epidemiology, Prevalence, Posterior longitudinal ligament, Ligamentum flavum, Diffuse idiopathic skeletal hyperostosis

## Abstract

**Background:**

The epidemiology and cause of ossification of the spinal ligaments (OSL) remains obscure. To date, there is no study that comprehensively evaluates the prevalence, distribution, and concomitance of each type of OSL by CT among general Chinese population. We therefore aimed to comprehensively investigate epidemiological characteristics of OSL using whole spine CT in the Chinese population and examine the factors that correlate with the presence of OSL.

**Methods:**

Ossification of the posterior longitudinal ligament (OPLL), ligamentum flavum (OLF), anterior longitudinal ligament (OALL), nuchal ligament (ONL), and diffuse idiopathic skeletal hyperostosis (DISH) were evaluated from the subjects who underwent PET/CT for the purpose of cancer screening in our hospital. Prevalence, distribution, and concomitance of OSL were reviewed. Logistic regression analysis was performed to identify the risk factors of OSL.

**Results:**

A total of 2000 subjects (1335 men and 665 women) were included. The prevalence rate of cervical OPLL (C-OPLL) was 4.1%, thoracic OPLL (T-OPLL) 2.25%, lumbar OPLL (L-OPLL) 0.8%, thoracic OLF (T-OLF) 37.65%, lumbar OLF (L-OLF) 1.45%, ONL 31.5%, DISH 3.85%. The most commonly involved level was C5 for C-OPLL, T1 for T-OPLL, T10 for T-OLF, and T8/9 for OALL. 21% of subjects with C-OPLL had T-OPLL, 44% of C-OPLL had T-OLF, 38% of T-OPLL had C-OPLL, 53% of T-OPLL had T-OLF, 44% of L-OPLL had T-OPLL, and 56% of L-OPLL had T-OLF. The average age of OSL-positive subjects was significantly higher than that of OSL-negative subjects. The results of the multiple regression analysis revealed that males had a strong association with DISH (odds ratio, 3.15; 95% confidence interval, 1.27–7.78; *P* = 0.013).

**Conclusion:**

The prevalence of OSL in the Chinese was revealed. Tandem ossification is not uncommon in people with OSL. There is a high incidence of multiple-regional OPLL in the whole spine. Approximately half of the subjects with OPLL coexist with T-OLF. For patients with clinical symptoms induced by OPLL, thorough evaluation of whole spine using CT is recommended.

## Background

Ossification of the spinal ligaments (OSL) is a pathologic condition characterized by heterotropic ossification of the spinal ligaments, such as ossification of the posterior longitudinal ligament (OPLL), ligamentum flavum (OLF), anterior longitudinal ligament (OALL), nuchal ligament (ONL) and diffuse idiopathic skeletal hyperostosis (DISH) [1, 2]. OPLL and OLF are common causes of spinal stenosis and spinal cord compression, which can cause various degrees of neurological symptoms [3, 4]. But many affected individuals are usually asymptomatic when the lesions are small [5]. DISH is a skeletal disease characterized by progressive ossification of the anterolateral side of the spine [6]. Although DISH is thought to be an asymptomatic condition in most affected individuals not aware of its presence, several clinical symptoms have been reported including pain, restriction of spinal movements, dysphagia at cervical level, and increased risk of unstable spinal fractures after trauma [7, 8]. To today, there have been several epidemiological investigations in the Far East Asian population, especially in Japanese. However, researches have been rarely conducted among Chinese population.

The epidemiology and etiology of OSL remains obscure. According to our review of the literature, there have been only three researches reporting the prevalence of OSL in the Chinese population [9–11]. Lang et al. [10] reported that the prevalence of thoracic OLF (T-OLF) was 63.9% in Chinese patients (*n* = 993) with chest symptoms using chest CT. Guo et al. [9] reported that the prevalence of T-OLF was 3.8% in Chinese individuals (*n* = 1736) using MRI. And it must be noted that their study population had a much younger average age of 38 years. Wang et al. [11] reported that the prevalence of ONL was 49.7% in Chinese patients (*n* = 372) with cervical spondylosis using plain radiographs and CT. Until now, no epidemiological study has assessed the prevalence of the OPLL, DISH, and OALL in Chinese population. And computed tomography (CT) may be the best modality for detecting the OSL, because it has a high resolution on density and can eliminate the influence of overlapping [12].

In light of this, the aim of this study was to comprehensively evaluate epidemiological characteristics of each type of OSL using whole body CT scans in the Chinese population and examine the factors that correlate with the presence of OSL.

## Methods

### Participants

From October 2010 to September 2013, a total of consecutive 2059 Chinese individuals from East China who underwent fluorin-18 fluorodeoxyglucose positron emission tomography and CT (PET/CT) for the purpose of cancer screening in our hospital were selected. Exclusion criteria were age of younger than 20 years or a history of spine surgery. If more than one CT scan were taken within the study period, the last examination was selected for the present study. As a result, 2000 subjects (1335 men and 665 women) were recruited for the analysis. Demographic data, including age, sex, height, weight and body mass index (BMI), were retrospectively reviewed. This study has been approved by our institutional ethics committee.

### Radiographic assessment

**Fig. 1:**
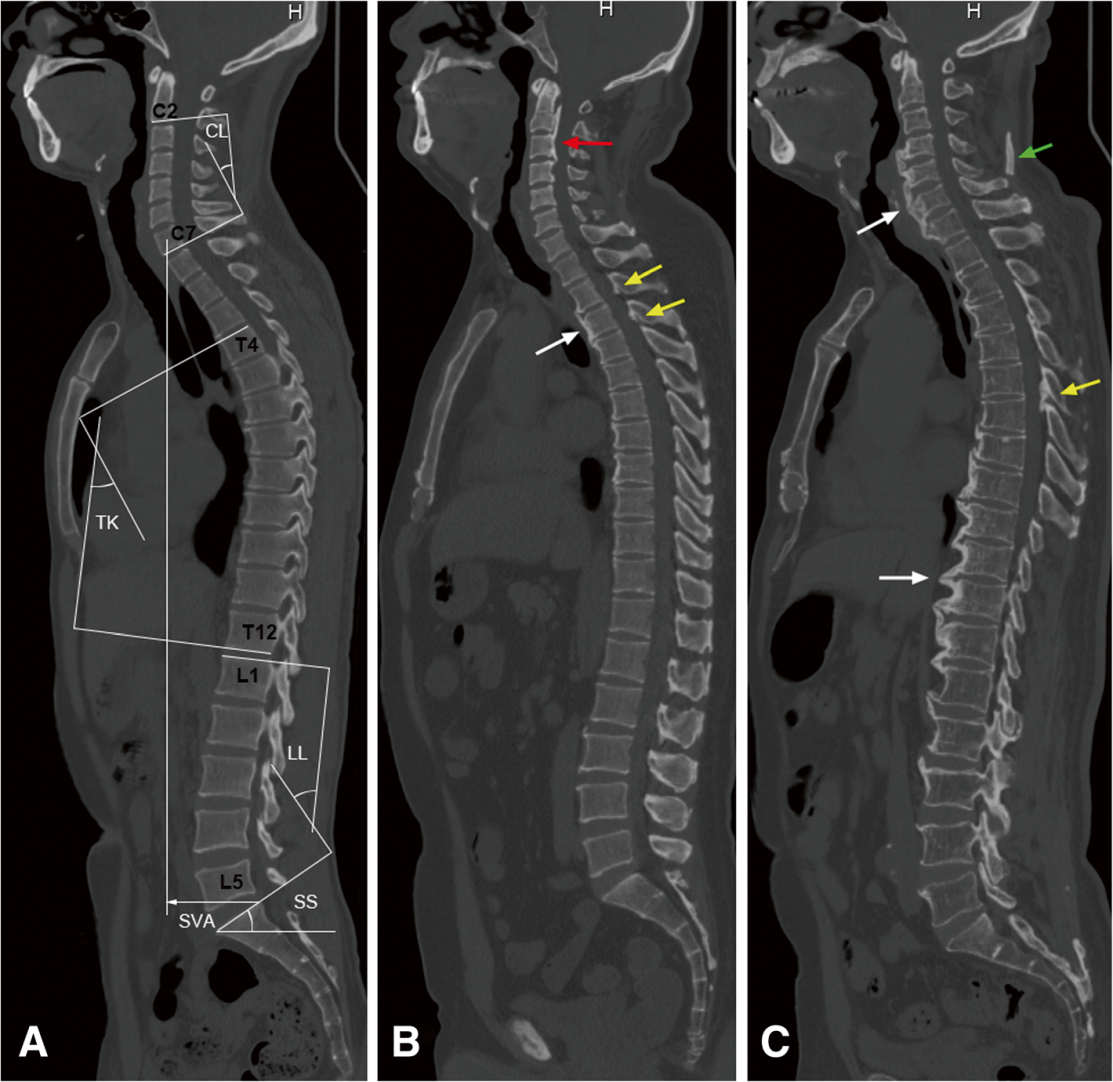
Sagittal radiologic parameters and examples of each type of OSL on whole spine computed tomography. **a** Measurement methods for CL, TK, LL, SS, and SVA. **b** A 50-year-old man had OPLL (red arrow), OLF (yellow arrow), and OALL (white arrow). **c** A 70-year-old man had OLF (yellow arrow), DISH (white arrow), and ONL (green arrow). Abbreviations: CL, cervical lordosis; TK, thoracic kyphosis; LL, lumbar lordosis; SS, sacral slope; SVA, sagittal vertical axis; OPLL, ossification of the posterior longitudinal ligament; OLF, ossification of the ligamentum flavum; OALL, ossification of the anterior longitudinal ligament; DISH, diffuse idiopathic skeletal hyperostosis; ONL, ossification of the nuchal ligament

To our knowledge, there is no universally agreed upon definition of OSL on CT. We therefore made a diagnosis of OSL according to previous researches with some modifications. Definitive OPLL and OLF were defined as the ossification, at least, thicker than 2 mm on axial CT scan [13–17]. OALL was defined as ossification thicker than 3 mm on axial CT scan and need to bridge the adjacent vertebrae [16, 18]. DISH was diagnosed according to the commonly used diagnostic criteria, defined by Resnick and Niwayama [19]. The criteria are as follows: (1) the presence of contiguous ligamentous ossification involving three or more intervertebral disk levels(4 or more consecutive fused vertebral bodies) with anterior or lateral bridging; (2) preserved intervertebral disc space; and (3) absence of apophyseal joint ankylosis and sacroiliac joint fusion (Fig. 1).

For statistics on the prevalence and distribution of OSL, if OPLL was located at C7/T1 or T12/L1 intervertebral level, they were included in the cervical and thoracic segments, respectively. Similarly, OALL, bridging the adjacent vertebrae at C7/T1 or T12/L1 intervertebral level, were included in the cervical and thoracic segments, respectively. Pelvic and spinal sagittal parameters were measured on the midline sagittal image (Fig. 1). Cervical lordosis (C2–7, CL), thoracic kyphosis (T4–12, TK), and lumbar lordosis (L1–S1, LL) were measured by Cobb method. Sacral slope (SS) was measured between the tangent line to the superior endplate of S1 and the horizontal line. Sagittal vertical axis (SVA) was the distance between the C7 plumb line and the posterosuperior corner of S1. Cobb angles measured from supine CT may be underestimated compared with standing radiograph Cobb measurements. But supine CT curve measurements are also valuable in biomechanical analysis, because the supine position provides an approximate “zero load” configuration for the spine [20].

All whole body CT scans were obtained on a PET/CT scanner (Discovery VCT, General Electric, Milwaukee, Wisconsin, USA) with the following settings: tube current 200 mA, tube voltage 140 kV, thickness 3.75 mm, collimation 64 × 0.6 mm, pitch 0.516, matrix 512 × 512, and gantry rotation time 0.33 s. Scanning was performed from head to mid-thigh in the supine position. Reconstructed axial and sagittal images were reviewed on a uWS-MI R001 workstation (United Imaging).

Images were reviewed in the following steps. Firstly, an orthopaedic spine surgeon and a radiologist independently evaluated 50 subjects from the enrolled cases. Disagreements were resolved by discussion with another senior orthopedic surgeon in a consensus meeting. Secondly, for testing the reliability of diagnoses, two observers independently evaluated another 100 subjects to assess the interobserver reliability. Once again, any differences were resolved by consensus. And then, the orthopaedic spine surgeon analyzed the 100 subjects again with a six weeks interval to assess the intraobserver reliability. Finally, the remaining subjects were reviewed by the orthopedic surgeon.

### Statistical analysis

The data were analyzed using IBM SPSS 22.0 statistical software (IBM SPSS Inc., Armonk, NY, USA). Categorical data analyzed using chi-square or Fisher’s exact tests. Measurement data analyzed using Student t test or Welch test. Logistic regression analysis was used to test the association between OSL and potential risk factors. Explanatory variables, such as age (+ 1 year), gender (0 = women, 1 = men), and height (+ 1 cm), identified by the univariate logistic regression analysis as potential risk factors were selected for inclusion in a multivariate logistic regression analysis. To ensure selection of the best combination of explanatory variables, only those with a *P* < 0.05 were included in the model.

Kappa analysis was performed to determine interobserver and intraobserver reliabilities. Kappa values above 0.81, 0.61–0.80, 0.41–0.60, 0.21–0.40, and 0–0.20 indicated almost perfect, substantial, moderate, fair and slight agreements, respectively [21]. For all analyses, statistical significance was set at a level of *P* < 0.05.

## Results

The Kappa value of inter- and intra-observer reliabilities were 0.71 and 0.90, respectively.

### Demographic data

A total of 2000 subjects were included, there were 1335 men and 665 women. Table 1 displays the demographic characteristics of the study population. The mean age of the subjects was 48.5 ± 9.9 years(range, 22 to 95 years), height was 167.4 ± 7.8 cm, body weight was 68.8 ± 12.6 kg, and BMI was 24.4 ± 3.3 kg/m^2^. The average regional Cobb angles were as follows: CL 7.9 ± 6.2°, TK 18.3 ± 7.7°, and LL 40.6 ± 10.5°. The mean value of SS and SVA were, respectively, 36.9 ± 7.5° and 18.7 ± 19.7 mm.

**Table 1:** Demographic data of the study subjects

Parameters	Total	Men	Women
Number	2000	1335	665
Age(y)	48.5 ± 9.9	48.3 ± 9.7	48.9 ± 10.2
Height(cm)	167.4 ± 7.8	171.4 ± 5.6	159.4 ± 5.2
Weight(kg)	68.8 ± 12.6	68.8 ± 12.6	58.1 ± 8.4
BMI(kg/m^2^)	24.4 ± 3.3	25.2 ± 3.2	22.9 ± 3.1
CL(°)	7.9 ± 6.2	7.8 ± 6.3	8.0 ± 6.1
TK(°)	18.3 ± 7.7	19.3 ± 7.7	16.5 ± 7.5
LL(°)	40.6 ± 10.5	40.1 ± 10.4	41.6 ± 10.8
SS(°)	36.9 ± 7.5	36.9 ± 7.3	37.0 ± 7.9
SVA(cm)	1.87 ± 1.97	2.32 ± 2.11	0.97 ± 1.23

*BMI* body mass index, *CL* cervical lordosis, *TK* thoracic kyphosis, *LL* lumbar lordosis, *SS* sacral slope, *SVA* sagittal vertical axiss

### Prevalence, distribution, and concomitance of OPLL

**Fig. 2:**
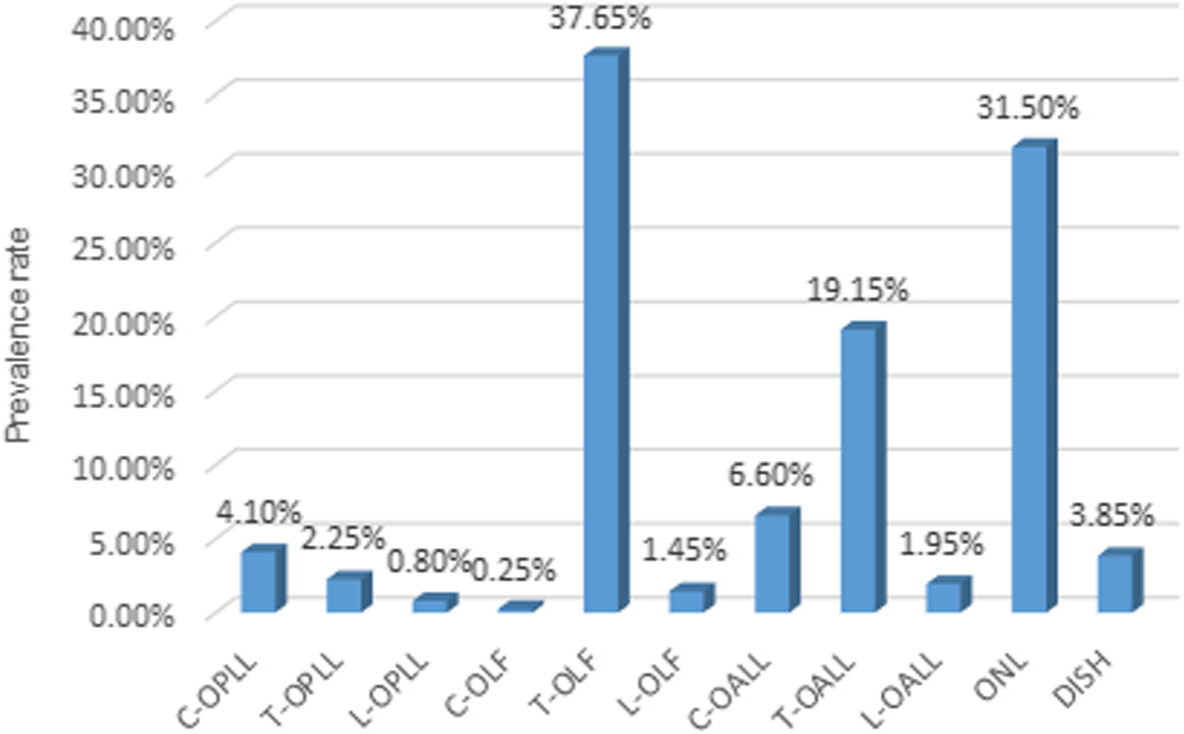
Prevalence of each type of spinal ligament ossification. Abbreviations: C, cervical; T, thoracic; L, lumbar; OPLL, ossification of the posterior longitudinal ligament; OLF, ossification of the ligamentum flavum; OALL, ossification of the anterior longitudinal ligament; ONL, ossification of the nuchal ligament; DISH, diffuse idiopathic skeletal hyperostosis

A total of 82 subjects had cervical OPLL (C-OPLL), including 55 men and 27 women. The prevalence of C-OPLL was 4.1% (men, 4.12%; women, 4.06%). A total of 45 subjects had thoracic OPLL (T-OPLL), including 21 men and 24 women. The prevalence of T-OPLL was 2.25% (men, 1.57%; women, 3.61%). A total of 16 subjects had lumbar OPLL (L-OPLL), including 10 men and 6 women. The prevalence of L-OPLL was 0.8% (men, 0.75%; women, 0.9%). The statistically significant difference between men and women was only in the prevalence of T-OPLL (*p* = 0.004) (Table 2, Fig. 2).

**Table 2:** Prevalence of each type of spinal ligament ossification

	Total	Prevalence (%)		
		Men	Women	P
C-OPLL	4.10 [3.23–4.97]	4.12 [3.05–5.19]	4.06 [2.56–5.56]	0.949
T-OPLL	2.25 [1.60–2.90]	1.57 [0.90–2.24]	3.61 [2.19–5.03]	0.004
L-OPLL	0.80 [0.41–1.19]	0.75 [0.29–1.21]	0.90 [0.18–1.62]	0.717
C-OLF	0.25 [0.03–0.47]	0.22 [0–0.48]	0.30 [0–0.72]	1.000
T-OLF	37.65 [35.52–39.78]	36.10 [33.53–38.68]	40.75 [37.01–44.50]	0.043
L-OLF	1.45 [0.93–1.97]	1.04 [0.50–1.60]	2.26 [1.12–3.39]	0.033
C-OALL	6.60 [5.51–7.69]	7.86 [6.42–9.31]	4.06 [2.56–5.56]	0.001
T-OALL	19.15 [17.42–20.88]	22.17 [19.94–24.40]	13.08 [10.51–15.65]	< 0.001
L-OALL	1.95 [1.34–2.56]	2.47 [1.64–3.31]	0.90 [0.18–1.62]	0.017
ONL	31.50 [29.46–33.54]	38.58 [35.96–41.19]	17.29 [14.41–20.18]	< 0.001
DISH	3.85 [3.01–4.69]	4.87 [3.71–6.02]	1.80 [0.79–2.82]	0.001

[]: 95% confidence interval

*C* cervical, *T* thoracic, *L* lumbar, *OPLL* ossification of the posterior longitudinal ligament, *OLF*, ossification of the ligamentum flavum, *OALL* ossification of the anterior longitudinal ligament, *ONL* ossification of the nuchal ligament, *DISH* diffuse idiopathic skeletal hyperostosis

**Fig. 3:**
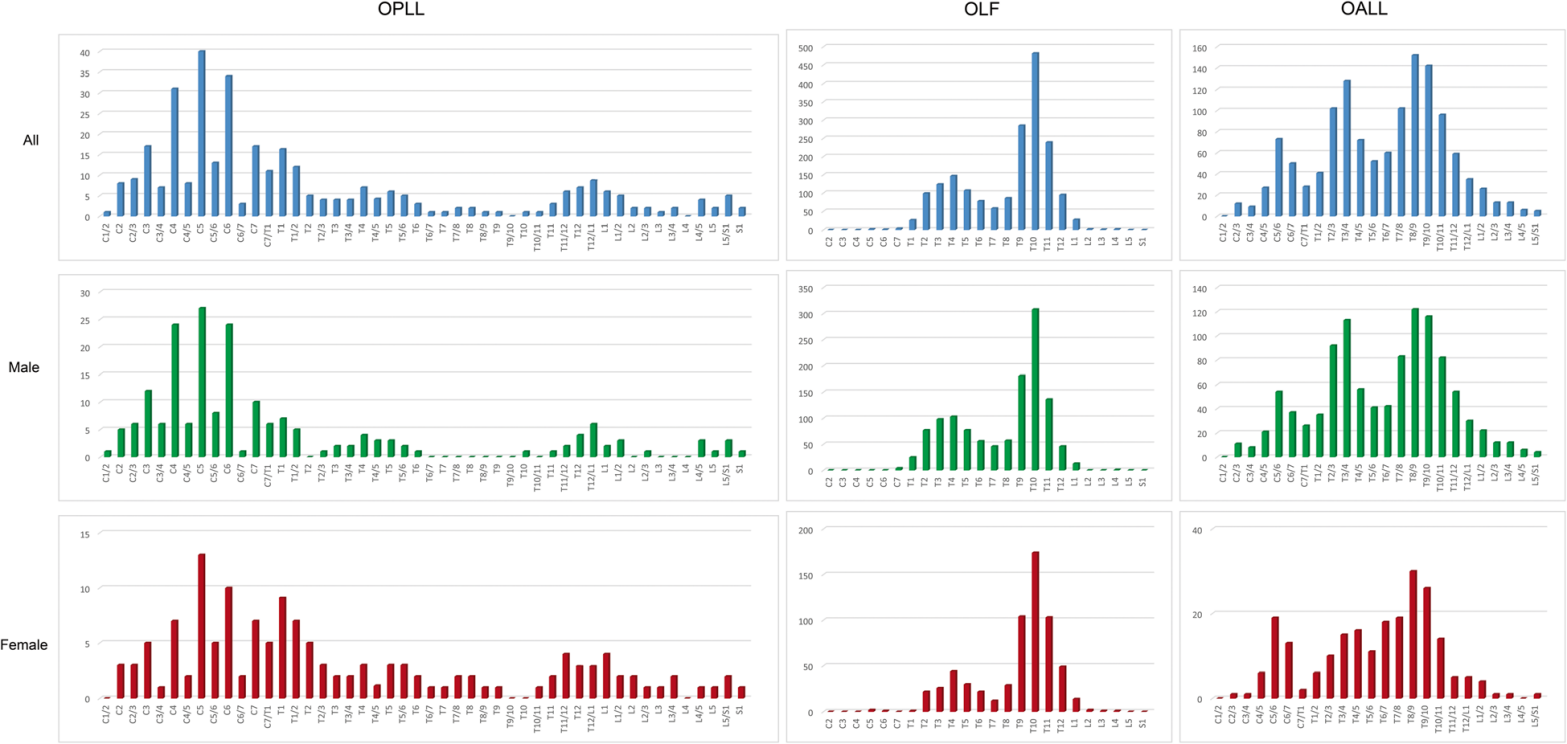
Distribution of the OPLL, OLF, and OALL between genders. Abbreviations: OPLL, ossification of the posterior longitudinal ligament; OLF, ossification of the ligamentum flavum; OALL, ossification of the anterior longitudinal ligament

Most of OPLL were located in the cervical spine (C2-C7/T1). The upper thoracic spine (T1-T6) and thoracolumbar junctional region (T11-L1/2) were less frequently involved (Fig. 3). The highest involvement of OPLL was most commonly detected at C5 (40 cases), followed by C6 (34 cases), C4 (31 cases), C3 (17 cases), C7 (17 cases), and T1 (16 cases). Figure 3 showed similar distribution between men and women in each region of the spine. However, the distribution of OPLL for women showed two peaks with the highest and second highest peak found at C5 and T1, respectively.

Of all cases of C-OPLL, 21% had T-OPLL, 4% had L-OPLL, 44% had T-OLF, 46% had ONL, 21% had cervical OALL (C-OALL), 52% had thoracic OALL (T-OALL), and 17% had DISH. Of all cases of T-OPLL, 38% had C-OPLL, 16% had L-OPLL, 53% had T-OLF, 22% had C-OALL, 60% had T-OALL, 40% had ONL, and 18% had DISH. Of all cases of L-OPLL, 19% had C-OPLL, 44% had T-OPLL, 56% had T-OLF, and 44% had ONL.

### Prevalence, distribution, and concomitance of OLF

A total of 5 subjects had cervical OLF (C-OLF), including 3 men and 2 women. The prevalence of C-OLF was 0.3% (men, 0.2%; women, 0.3%). A total of 753 subjects had T-OLF, including 482 men and 271 women. The prevalence of T-OLF was 37.7% (men, 36.1%; women, 40.8%). A total of 29 subjects had lumbar OLF (L-OLF), including 14 men and 15 women. The prevalence of L-OLF was 1.5% (men, 1.0%; women, 2.3%). The differences between men and women were statistically significant in the prevalence of T-OLF and L-OLF (*p* < 0.05). There was no significant difference in the prevalence of C-OLF between men and women (*p* = 1.000) (Table 2, Fig. 2).

Most of OLF were located in the thoracic spine. The distribution of T-OLF formed two peaks with the highest and second highest peak found at T10 and T4, respectively (Fig. 3). T-OLF was found mostly at T10 (482 cases), followed by T9 (285 cases), and T11 (239 cases). Figure 3 showed similar distribution between men and women.

Of all cases of T-OLF, 5% had C-OPLL, 3% had T-OPLL, 4% had L-OLF, 25% had T-OALL, 36% had ONL, and 6% had DISH.

### Prevalence, distribution, and concomitance of OALL

A total of 132 subjects had C-OALL, including 105 men and 27 women. The prevalence of C-OALL was 6.6% (men, 7.9%; women, 4.1%). A total of 383 subjects had T-OALL, including 296 men and 87 women. The prevalence of T-OALL was 19.2% (men, 22.2%; women, 13.1%). A total of 39 subjects had lumbar OALL (L-OALL), including 33 men and 6 women. The prevalence of L-OALL was 2% (men, 2.5%; women, 0.9%). Statistical analysis showed that the prevalence of C-OALL, T-OALL, and L-OALL were significantly higher among the males (*P* < 0.05) (Table 2, Fig. 2).

Most of OALL were located in the thoracic spine. Three of the most commonly affected levels were T8/9 (152 cases), T9/10 (142 cases), and T3/4 (128 cases). The distribution of T-OALL showed two peaks in men but only one peak in women (Fig. 3).

Of all cases of C-OALL, 13% had C-OPLL, 62% had ONL, 56% had T-OLF, 69% had T-OALL, and 30% had DISH. Of all cases of T-OALL, 7% had T-OPLL, 50% had T-OLF, 11% had C-OPLL, 24% had C-OALL, 51% had ONL, and 20% had DISH.

### Prevalence and concomitance of ONL and DISH

A total of 630 subjects had ONL, including 515 men and 115 women. The prevalence of ONL was 31.5% (men, 38.6%; women, 17.3%). A total of 77 subjects had DISH, including 65 men and 12 women. The prevalence of DISH was 3.9% (men, 4.9%; women, 1.8%). The prevalence of ONL and DISH was significantly higher in males compared to females (*P* < 0.01) (Table 2, Fig. 2).

Of all cases of ONL, 6% had C-OPLL, 13% had C-OALL, 43% had T-OLF, 31% had T-OALL, and 8% had DISH. Of all cases of DISH, 18% had C-OPLL, 10% had T-OPLL, 57% had T-OLF, and 65% had ONL.

### Clinical factors correlated with spinal ligament ossification

**Fig. 4:**
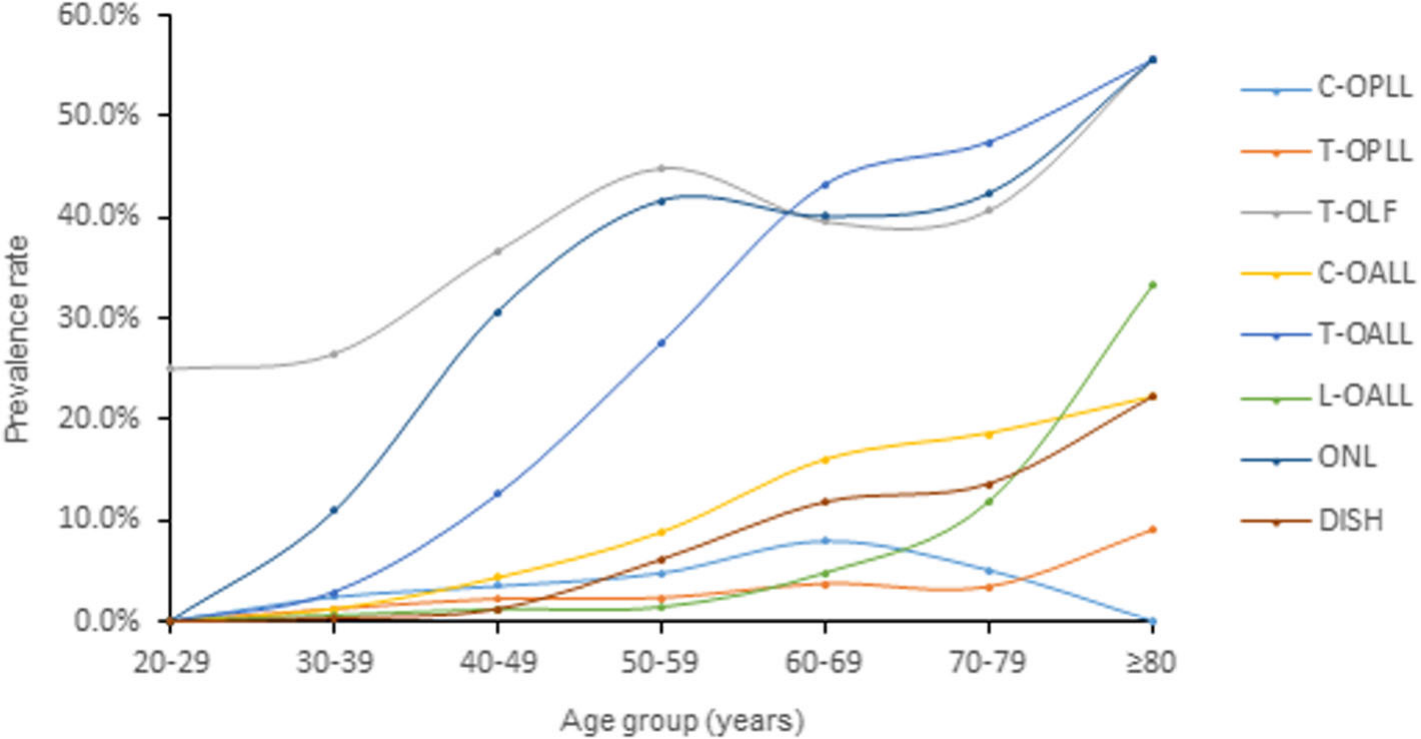
The prevalence of spinal ligament ossification according to each decade of individuals’ life. Abbreviations: C, cervical; T, thoracic; L, lumbar; OPLL, ossification of the posterior longitudinal ligament; OLF, ossification of the ligamentum flavum; OALL, ossification of the anterior longitudinal ligament; ONL, ossification of the nuchal ligament; DISH, diffuse idiopathic skeletal hyperostosis

We compared the prevalence of OSL among each 10-year age group (Fig. 4). The prevalence revealed an increase trend in older age group. The average age of OSL-positive subjects was significantly higher than that of OSL-negative subjects (Tables 3 and 4).

**Table 3:** Baseline characteristics of participants classified by the presence or absence of ossification

Number	C-OPLL			T-OPLL			T-OLF		
	+(*n* = 82)	-(*n* = 1918)	p	+(*n* = 45)	-(*n* = 1955)	p	+(*n* = 753)	-(*n* = 1247)	p
Age(y)	51.7 ± 9.1	48.4 ± 9.9	0.003	51.8 ± 11.2	48.4 ± 9.9	0.026	49.7 ± 9.7	47.8 ± 9.9	< 0.001
Height(cm)	167.3 ± 7.6	167.4 ± 7.9	0.941	166.1 ± 7.9	167.4 ± 7.8	0.259	167.1 ± 7.7	167.6 ± 7.9	0.257
Weight(kg)	74.6 ± 13.8	68.5 ± 12.5	< 0.001	71.5 ± 13.6	68.7 ± 12.6	0.156	68.3 ± 12.9	69.1 ± 12.4	0.222
BMI(kg/m^2^)	26.5 ± 3.8	24.3 ± 3.3	< 0.001	25.8 ± 3.3	24.4 ± 3.3	0.008	24.3 ± 3.4	24.5 ± 3.3	0.364
CL(°)	8.2 ± 7.1	7.8 ± 6.2	0.648	8.5 ± 8.2	7.8 ± 6.2	0.479	8.0 ± 6.3	7.8 ± 6.2	0.380
TK(°)	16.8 ± 7.0	18.4 ± 7.7	0.073	19.1 ± 8.8	18.3 ± 7.7	0.505	19.2 ± 8.1	17.8 ± 7.4	< 0.001
LL(°)	38.9 ± 9.1	40.7 ± 10.6	0.128	41.2 ± 11.1	40.6 ± 10.5	0.708	41.8 ± 10.7	39.9 ± 10.3	< 0.001
SS(°)	35.9 ± 6.6	37.2 ± 10.7	0.293	37.4 ± 7.6	37.1 ± 10.6	0.853	37.5 ± 7.7	36.6 ± 7.4	0.008
SVA(cm)	1.79 ± 1.10	1.88 ± 2.00	0.716	1.84 ± 1.30	1.87 ± 1.99	0.916	1.86 ± 1.33	1.88 ± 2.28	0.811

*BMI* body mass index, *CL* cervical lordosis, *TK* thoracic kyphosis, *LL* lumbar lordosis, *SS* sacral slope, *SVA* sagittal vertical axis, *C* cervical, *T* thoracic, *L* lumbar, *OPLL* ossification of the posterior longitudinal ligament, *OLF* ossification of the ligamentum flavum

**Table 4:** Baseline characteristics of participants classified by the presence or absence of ossification

Number	T-OALL			ONL			DISH		
	+(*n* = 383)	-(*n* = 1617)	p	+(*n* = 630)	-(*n* = 1370)	p	+(*n* = 77)	-(*n* = 1923)	p
Age(y)	55.2 ± 9.6	46.9 ± 9.3	< 0.001	51.7 ± 9.2	47.0 ± 9.8	< 0.001	58.7 ± 9.4	48.1 ± 9.7	< 0.001
Height(cm)	167.7 ± 7.6	167.3 ± 7.9	0.356	169.2 ± 7.0	166.6 ± 8.1	< 0.001	167.7 ± 7.5	167.4 ± 7.9	0.753
Weight(kg)	72.1 ± 11.7	68.0 ± 12.7	< 0.001	73.1 ± 11.8	66.7 ± 12.5	< 0.001	73.7 ± 13.5	68.6 ± 12.5	0.003
BMI(kg/m^2^)	25.5 ± 3.1	24.1 ± 3.3	< 0.001	25.4 ± 3.2	23.9 ± 3.3	< 0.001	26.1 ± 3.7	24.3 ± 3.3	< 0.001
CL(°)	8.9 ± 7.4	7.6 ± 5.9	0.002	7.8 ± 6.2	7.9 ± 6.2	0.879	10.9 ± 8.4	7.7 ± 6.1	0.001
TK(°)	22.2 ± 9.6	17.4 ± 6.9	< 0.001	19.2 ± 8.0	17.9 ± 7.5	< 0.001	24.4 ± 9.6	18.1 ± 7.5	< 0.001
LL(°)	43.4 ± 10.4	40.0 ± 10.4	< 0.001	40.3 ± 10.6	40.8 ± 10.5	0.399	43.5 ± 9.9	40.5 ± 10.5	0.013
SS(°)	38.2 ± 7.4	36.8 ± 11.1	0.027	36.6 ± 7.3	37.3 ± 11.7	0.148	37.9 ± 7.1	37.1 ± 10.6	0.511
SVA(cm)	2.12 ± 1.30	1.81 ± 2.10	0.006	2.07 ± 2.61	1.78 ± 1.59	0.002	2.38 ± 1.4	1.85 ± 1.99	0.022

*BMI* body mass index, *CL* cervical lordosis, *TK* thoracic kyphosis, *LL* lumbar lordosis, *SS* sacral slope, *SVA* sagittal vertical axis, *T* thoracic, *OALL* ossification of the anterior longitudinal ligament, *ONL* ossification of the nuchal ligament, *DISH* diffuse idiopathic skeletal hyperostosis

C-OPLL-positive individuals had significantly higher weight and BMI than C-OPLL-negative (*P* < 0.001). T-OLF-positive individuals had significantly higher TK, LL, and SS than T-OLF-negative (*P* < 0.01). ONL-positive individuals had significantly higher BMI and SVA than ONL-negative (*P* < 0.01). Individuals with T-OALL or DISH had significantly higher spinal sagittal parameters and BMI than those without. (Tables 3 and 4).

**Fig. 5:**
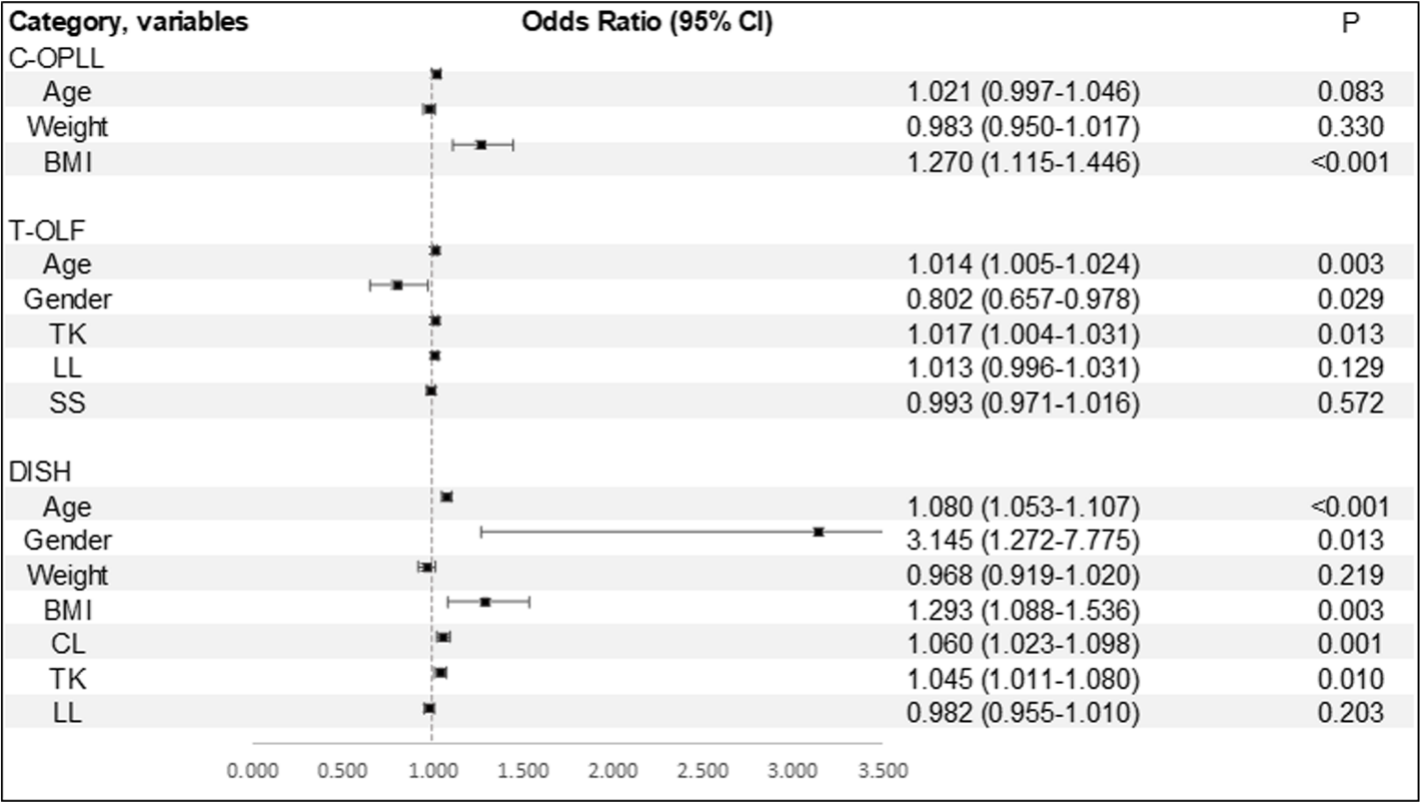
Estimated associations (odds ratio [OR] and 95% confidence interval) of selected demographic and clinical factors with spinal ligament ossification: results from multivariable logistic regression analyses. Abbreviations: BMI, body mass index; CL, cervical lordosis; TK, thoracic kyphosis; LL, lumbar lordosis; SS, sacral slope

Figure 5 demonstrates the result of multivariate logistic regression analysis. Males showed a strong association with DISH (odds ratio, 3.15; 95% confidence interval, 1.27–7.78; *P* = 0.013). BMI was found to be significantly associated with the presence of C-OPLL and DISH. In addition, increased age and TK were also found to be significant associated factors for the presence of T-OLF and DISH.

## Discussion

The present study revealed the prevalence of OSL in the Chinese population. The prevalence rate of C-OPLL was 4.1%, T-OPLL 2.25%, L-OPLL 0.8%; C-OLF 0.25%, T-OLF 37.65%, L-OLF 1.45%; C-OALL 6.6%, T-OALL 19.15%, L-OALL 1.95%; ONL 31.5%; DISH 3.85%. To the best of our knowledge, this study is the first to comprehensively examine the prevalence of each type of OSL, using whole-body CT, in the Chinese population and evaluate the factors that correlate with the presence of OSL.

OPLL and OLF could be a latent cause of neurologic symptoms induced by spinal cord compression. According to previous reports, the prevalence of C-OPLL was 1.9 to 6.3% in Japanese [16, 18, 22–24], 0.6 to 5.7% in Korean [25, 26], 4.8% in Asian Americans [15], and 0.7 to 1.3% in Caucasian [15, 27]. The prevalence of T-OLF was 3.6 to 36% in Japanese [16, 28–30], 3.8 to 63.9% in Chinese [9, 10], and 16.9 to 21.8% in Korean [4, 31]. The prevalence of T-OPLL was 0.56 to 1.9% in Japanese [16, 30, 32]. Our CT-based study showed that the prevalence was 4.1% in C-OPLL, 37.65% in T-OLF, and 2.25% in T-OPLL. It was found that the prevalence results of this study in Chinese were roughly consistent with other eastern Asians. And the prevalence of C-OPLL in western Caucasians is relatively lower than in eastern Asians, suggesting that genetic or ethnic factor could be related with the onset of OPLL. High prevalence of T-OLF was found among eastern Asians. Few studies have assessed the prevalence of T-OLF among western Caucasians. Although T-OLF has been considered unusual among Caucasians [9, 29], Williams et al. [33] reported the prevalence of T-OLF was 26% in 100 western Caucasians using CT. However, due to the limitation in the number of studies and sample size, it is difficult to compare the prevalence of OLF between eastern Asians and western Caucasians. Future studies should address these limitations.

One must note that if we classified the above-mentioned prevalence by diagnostic modality, we could find the prevalence of OSL by CT scan was generally higher than that of previous reports using plain radiographs or MRI. These diverse results show that the diagnostic modality has a significant impact on the assessment of OSL. Plain radiograph is not sensitive enough to detect small ossifications. Because C-OPLL in the lower cervical spine is likely to be masked by shoulder girdle shadows [14, 26]. T-OLF and T-OPLL are likely to be masked by superimposed bony structures such as ribs [29, 34]. MRI is also less sensitive for identifying small ossifications and thickened or folded ligamentum flavum [4, 10]. CT has been shown to have higher sensitivity for identifying OSL and is more likely to discover the actual prevalence of OSL [14, 16, 26, 35].

Several reports have shown that tandem ossification is not uncommon in people with OSL [16, 36, 37]. Hirai et al. [36] and Kawaguchi et al. [37] revealed that more than half of the patients with neurological symptoms caused by C-OPLL had coexisting OPLL in the thoracolumbar spine. Fujimori et al. [16] reported that more than half of the individuals with T-OPLL also had C-OPLL and 46% of T-OPLL also had T-OLF among general Japanese population. Similarly, in this study, we found that 21% of subjects with C-OPLL had T-OPLL, 44% of C-OPLL had T-OLF, 38% of T-OPLL had C-OPLL, 53% of T-OPLL had T-OLF, 19% of L-OPLL had C-OPLL, 44% of L-OPLL had T-OPLL, and 56% of L-OPLL had T-OLF among Chinese population. It can be found that subjects with OPLL generally have a predisposition to coexist with multiple-regional lesions. There is a high incidence of multiple-regional OPLL in the whole spine. In addition, it should be noted that approximately half of the subjects with OPLL coexist with T-OLF. Missed multiple-regional lesions can lead to serious consequences. Takeuchi et al. [38] reported case reports of thoracic paraplegia due to missed thoracic compressive lesion developing after a routine lumbar laminectomy. We suggest that it is not necessary to use whole spine CT as a routine screening. But, for patients with clinical symptoms induced by OPLL, we recommend thorough evaluation of whole spine using CT.

Tables 5 and 6 give a summary of studies performed by various authors on the prevalence of OSL by sex. Our data showed that T-OPLL is significantly more common in women, while DISH is significantly more common in men. The results of the multiple regression analysis revealed that males are three times more likely to suffer from DISH. Consistent with previous evidence [6, 16, 32, 40, 41, 43–49], female preponderance of T-OPLL and male preponderance of DISH were confirmed. With regard to the gender difference in the prevalence of C-OPLL, our current study showed that the prevalence of C-OPLL in men is almost equal to that in women. (men 4.12%, women 4.06%). C-OPLL prevalence rate of men in our study is far lower than that of previous studies using CT scan [16, 26]. In Japan and Korea, the prevalence has been considered to have a male predominance of roughly 2:1 to 3:1 [50]. Although eastern Asians are thought to be genetically similar, the discrepancy could be attributed to different reasons, such as age distribution, sex ratio, sample size, target population, or selection bias. In addition, lifestyle factors and dietary habits, including bad sleeping habits [51], high-salt and low-protein diet [52], were associated with an increased risk of OPLL. Some studies have shown that the coexistence of other disorders such as obesity [53], diabetes mellitus [54], hypoparathyroidism [5], and hormonal imbalance [55], are contributory factors in OPLL. Therefore, these multiple factors may lead to different results. For T-OLF, through a review of literature, we encountered 6 epidemiological studies reporting the prevalence of T-OLF. But the results regarding the gender difference in T-OLF prevalence are inconsistent. Four studies [4, 10, 16, 29] showed that T-OLF occurs predominantly in men, while two others [9, 31] showed the opposite result. In this study, T-OLF was significantly more common in women (men 36.10%, women 40.75%). Additional large-scale, multi-center studies is therefore necessary to confirm the cause for these differences.

**Table 5:** Previously reported prevalence of C-OPLL and T-OPLL

Type	Authors/reported year	Country	Race	Sample Size	Modality	Prevalence rate		
						M (%)	F (%)	T (%)
C-OPLL	Firooznia et al. [39]/1984	USA	White	1000	Cervical x-ray	NA	NA	0.7
	Ohtsuka et al. [23]/1987	Japan	Asian	1058	x-ray	4.3	2.4	3.2
	Shingyouchi et a [18]/1996	Japan	Asian	4802	Cervical x-ray	NA	NA	4.1
	Kim et al. [25]/2008	Korea	Asian	11,774	Cervical x-ray	0.79	0.45	0.6
	Yoshimura et al. [24]/2014	Japan	Asian	1562	Cervical x-ray	3.2	1.3	1.9
	Sohn et al. [26]/2014	Korea	Asian	3240	Thyroid CT	8.8	4.2	5.7
	Fujimori et al. [15]/2015	USA	White	1593	Cervical CT	1.6	0.8	1.3
			Asian	624	Cervical CT	5.8	3.6	4.8
			Hispanic	472	Cervical CT	1.5	3.1	1.9
			Aframerican	326	Cervical CT	2.2	2.0	2.1
	Fujimori et al. [16]/2016	Japan	Asian	1500	PETCT	8.3	3.4	6.3
	Present study	China	Asian	2000	PETCT	4.12	4.06	4.10
T-OPLL	Ono et al. [32]/1982	Japan	Asian	8610	Chest x-ray	0.25	0.74	0.56
	Ohtsuka et al. [30]/1986	Japan	Asian	1058	x-ray	0.9	0.6	0.8
	Mori et al. [40]/2014	Japan	Asian	3013	Chest CT	1.0	3.0	1.9
	Fujimori et al. [16]/2016	Japan	Asian	1500	PETCT	1.4	2.0	1.6
	Present study	China	Asian	2000	PETCT	1.57	3.61	2.25

*T* total, *M* male, *F* female, *C-OPLL* ossification of the posterior longitudinal ligament of the cervical spine, *T-OPLL* ossification of the posterior longitudinal ligament of the thoracic spine, *CT* computed tomography, *NA* not available, *PETCT* positron emission tomography and computed tomography

**Table 6:** Previously reported prevalence of T-OLF and DISH

Type	Authors/reported year	Country	Race	Sample Size	Modality	Prevalence rate		
						M (%)	F (%)	T (%)
T-OLF	Guo et al. [9]/2010	China	Asian	1736	MRI, CT	2.1	4.87	3.8
	Mori et al. [29]/2013	Japan	Asian	3013	Chest CT	38	33.9	36
	Lang et al. [10]/2013	China	Asian	993	Chest CT	68.5	59	63.9
	Moon et al. [31]/2015	Korea	Asian	2134	MRI	13.7	19	16.9
	Fujimori et al. [16]/2016	Japan	Asian	1500	PETCT	15	7.7	12
	Kim et al. [4]/2018	Korea	Asian	4999	Chest CT	23	20.1	21.8
	Present study	China	Asian	2000	PETCT	36.10	40.75	37.65
DISH	Julkunen et al. [41]/1975	Finland	White	8993	Chest x-ray	3.8	2.6	2.6
	Cassim et al. [42]/1990	South Africa	African	1500	Chest x-ray	3.8	4.2	3.9
	Weinfeld et al. [43]/1997	USA	Mixed race	2364	Chest x-ray	25	15	NA
	Kiss et al. [44]/2002	Hungary	White	635	x-ray	27.3	12.8	19.8
	Kim et al. [45]/2004	Korea	Asian	3595	Chest x-ray	5.4	0.8	2.9
	Westerveld et al. [46]/2008	Netherlands	White	501	Chest x-ray	22.7	12.1	17
	Kagotani et al. [6]/2015	Japan	Asian	1647	Whole spine x-ray	22	4.8	11
	Hirasawa et al. [47]/2016	Japan	Asian	558	Chest-pelvis CT	38.7	13.9	27.2
	Fujimori et al. [16]/2016	Japan	Asian	1500	PETCT	16	6.2	12
	Mori et al. [48]/2017	Japan	Asian	3013	Chest CT	13	2.5	8.7
	Hiyama et al. [49]/2018	Japan	Asian	1479	Whole spine CT	21.1	16	19.5
	Present study	China	Asian	2000	PETCT	4.87	1.80	3.85

*T* total, *M* male, *F* female, *T-OLF* ossification of the ligamentum flavum of the thoracic spine, *DISH* diffuse idiopathic skeletal hyperostosis, *CT* computed tomography, *NA* not available, *PETCT*, positron emission tomography and computed tomography, *MRI* magnetic resonance imaging

Our study showed the mean age of people with ossifications was significantly higher than those without. OSL is more prevalent in the older age group. We found that increasing age was significantly associated with the presence of T-OLF or DISH. This finding suggests that degeneration factor might affect the development of OSL. By multiple logistic regression analysis, we found that TK was significantly related to the presence of T-OLF and DISH. Similarly, Kim et al. [4] reported that people with T-OLF had significantly higher TK than others and believed that this suggested T-OLF was associated with mechanical stress. Because thoracic spine with greater TK often accompanied by higher tensile force. In the present study, T-OLF most frequently located in lower thoracic segments (T9–T12) and the second most frequent location was the upper thoracic segments (T2–T5). T-OPLL most frequently located in cervicothoracic junction region (T1–T2). Several studies considered that these locations are transitional areas in terms of spinal curvature, where is the area of high stress concentration [2, 4, 9, 10]. Therefore, it is possibly more prone to degeneration because of the high tensile forces.

The present study has several limitations. First, the study population was not randomly selected and not purely based on the general population. All individuals were collected in a tertiary, multi-specialty referral hospital, which inevitably creates a sample selection bias. However, it is considered unethical to perform whole body CT for normal volunteers due to the radiation exposure. Second, there are 59 subjects (3.0%) might suffer from cancer in the study sample. Incidence of cancer in our study was higher than the real cancer morbidity [56]. But through statistical analysis, we found that there is no significant difference in the prevalence of spinal ligament ossification between cancer-positive subjects and cancer-negative subjects (data not shown). Third, there is no information regarding the clinical presentation of OSL in this screening population. We could not evaluate the association between OSL and related clinical manifestations. Fourth, spinal sagittal parameters measured from supine position may be underestimated compared with standing position. In addition, OSL can have an influence on the flexibility of the spinal column, so there may be a limitation for measuring spinal sagittal parameters in the supine position. Nevertheless, considering the difficulty in obtaining whole body CT data in a large general population, we think that our data, to some extent, reflects the prevalence of OSL in the general population of China.

## Conclusions

The prevalence of spinal ligament ossifications in Chinese was revealed and roughly consistent with other eastern Asians. Tandem ossification is not uncommon in people with spinal ligament ossifications. There is a high incidence of multiple-regional OPLL in the whole spine. Approximately half of the subjects with OPLL coexist with T-OLF. For patients with clinical symptoms induced by OPLL, thorough evaluation of whole spine using CT is recommended.

## Abbreviations

BMI: Body mass index
C: Cervical
CL: Cervical lordosis
CT: Computed tomography
DISH: Diffuse idiopathic skeletal hyperostosis
L: Lumbar
LL: Lumbar lordosis
MRI: Magnetic resonance imaging
OALL: Ossification of the anterior longitudinal ligament
OLF: Ossification of the ligamentum flavum
ONL: Ossification of the nuchal ligament
OPLL: Ossification of the posterior longitudinal ligament
OSL: Ossification of the spinal ligaments
SS: Sacral slope
SVA: Sagittal vertical axis
T: Thoracic
TK: Thoracic kyphosis
